# Delusion of Pregnancy: An Updated Systematic Review

**DOI:** 10.3390/jcm15197403

**Published:** 2026-09-23

**Authors:** Matteo Angelo Fabris, Dawid Konrad Ścigała

**Affiliations:** 1 Department of Psychology, University of Turin, Via Verdi 10, 10124 Turin, Italy; 2 Department of Psychology, Institute of Psychology, The Maria Grzegorzewska University, Ul. Szczęśliwicka 40, 02-353 Warsaw, Poland; dscigala@aps.edu.pl

**Keywords:** delusion of pregnancy, delusional disorder, systematic review, geriatric psychosis, hyperprolactinaemia, delusional procreation syndrome

## Abstract

Delusion of pregnancy (DoP) is a rare psychopathological symptom characterised by the fixed, delusional conviction of being pregnant in the absence of objective evidence. The systematic review by Bera and Sarkar remains, to this day, the principal quantitative reference on the phenomenon, based on 84 cases published up to August 2014. The aim of the present work is to update this case series by including literature published between September 2014 and August 2026, and to analyse any clinical and aetiological differences that emerge relative to the original sample. Thirty-two new cases were identified from 18 publications, extracted using the same categorical grid employed in the original study and integrated with the 84 historical cases (combined sample, *n* = 116). All comparisons between the original and updated subsamples reported below are descriptive and exploratory; no inferential statistical testing was performed, and the reported percentages should not be interpreted as validated effect sizes. The general profile of the disorder—female predominance, schizophrenia as the most frequent underlying diagnosis, monofetal presentation, good overall treatment response—is substantially confirmed. The most marked differences between the two subsamples concern a higher proportion, among recently published cases, of organic/geriatric presentations (28.1% vs. 4.8%) and coenaesthopathic processes (43.8% vs. 20.2%); however, sensitivity analysis indicated that both differences are substantially attenuated (to 14.8% and 33.3%, respectively) once a single five-case series is excluded, suggesting that publication-level clustering, rather than a genuine population-level temporal trend, plausibly accounts for much of this apparent shift. The data suggest, on an exploratory basis, the clinical usefulness of distinguishing a primary form, of complex delusional origin, from a secondary form due to somatic misinterpretation—a distinction presented here as a hypothesis-generating proposal, not as a validated clinical typology, with potential implications for the diagnostic assessment pathway and for pharmacological and psychotherapeutic treatment approaches, to be confirmed through prospective, controlled study designs.

## 1. Introduction

Parenthood and reproduction are among the most intimate and important themes for human beings [1]. Delusion of pregnancy (DoP) is defined as a persistent, false conviction of being pregnant, maintained by an individual despite contrary factual evidence [2]. The Diagnostic and Statistical Manual of Mental Disorders, Fifth Edition (DSM-5), classifies it as a somatic subtype of delusional disorder when it presents in isolated form, within the spectrum of schizophrenia and other psychotic disorders [3].

DoP has ancient historical roots, with early accounts attributed to Hippocrates and a reported peak incidence of up to one case in every 250 pregnancies in the immediate post-war period, plausibly reflecting the intense socio-cultural pressures on motherhood of that era [4]. The disorder is typically considered rare, though population-level epidemiological data are lacking [2]; current knowledge, based on published cases, indicates a strong female predominance in the 20–40 age range, with male cases very rare [2,5].

DoP must be distinguished from other morbid conditions, in particular from pseudocyesis, which represents the main differential diagnosis. Pseudocyesis, also known as “phantom pregnancy,” differs from DoP in that it is characterised by the presence of objective signs of pregnancy—amenorrhoea, abdominal distension, breast engorgement, nausea—in the absence of a confirmed pregnancy [4,6]. More specifically, while DoP is conceptualised by current nosography as a somatic-type delusional disorder within the schizophrenia spectrum, pseudocyesis is defined as a psychosomatic disorder [3,7]. Further conditions to be differentiated are simulated pregnancy (a conscious falsification of the state of pregnancy) [8] and couvade syndrome, in which it is the male partner who manifests pseudo-pregnancy symptoms concurrently with his partner’s actual pregnancy [9].

A further diagnostic distinction, conceptually opposite but clinically adjacent, is represented by the phenomenon of denial of pregnancy, in which a woman who is actually pregnant does not recognise, consciously or unconsciously, her own condition [8]. Kenner and Nicolson [10], in their review of psychosomatic disorders of gravida status, propose a classification of denial of pregnancy and underline its clinical and medico-legal relevance. Indeed, if this condition goes unrecognised, the woman may find herself managing an unexpected, unassisted delivery, which also raises the risk of a fatal act against the newborn [11]. The authors define it as the “somatic inverse” of false pregnancy, emphasising how the two phenomena, although opposite in presentation, share a common psychosomatic matrix [10].

The diagnostic process for DoP is greatly complicated by the syndrome’s high rate of comorbidity with other disorders, particularly schizophrenia, bipolar disorder, and depression, as well as organic disorders such as epilepsy, hyponatraemia, hypothyroidism, metabolic syndrome, and urinary tract infections [7]. When DoP presents as an isolated symptom rather than in the context of established schizophrenia, it falls under the broader category of delusional disorder (DD). González-Rodríguez and Seeman [12], in their review of clinical management approaches for DD, note that delusional content tends to show gender differences: delusions of pregnancy and denial of pregnancy are typically found in women, while other subtypes (such as Koro syndrome) are predominantly found in men. Of particular relevance to the objectives of this update is the study by González-Rodríguez et al. [13], which specifically examined the presentation of delusional disorder in older adults. The authors hypothesise that delusional disorder becomes relatively more prevalent in older adults, especially among women, and that this prevalence is associated with demonstrable brain changes, which in turn are linked to cognitive deficits and a less favourable response to medication. In particular, the cognitive dimension of the disorder appears to be associated with non-prominent hallucinations, poorer overall functioning, and greater somatic comorbidity—an association that becomes more pronounced with increasing age. This hypothesis, formulated on a general basis for delusional disorder, provides a direct theoretical framework for interpreting the findings of this review specifically with regard to delusion of pregnancy.

The primary quantitative reference on DoP remains, to this day, the systematic review by Bera and Sarkar [5], which has the merit of having systematised 84 cases published in 40 articles up to August 2014, deriving a reference demographic, clinical, and aetiological profile through a structured data-extraction form. A second significant contribution, although focused specifically on organic comorbidities, is the systematic review by Gogia et al. [14] of 140 cases of DoP, which identified an associated medical or surgical condition in 23 cases, with symptom resolution in 10 out of 15 cases that underwent targeted medical intervention. However, despite these contributions, the literature lacks any other attempt to systematically organise DoP cases with the aim of identifying recurring characteristics and potential risk factors. An update is warranted not merely because a further eleven years of case reports have accumulated, but because a substantially enlarged pooled sample is needed to determine whether the demographic, diagnostic, clinical, and aetiological profile described in 2015 remains representative of the published literature, or whether specific presentations—such as the organic and geriatric cases that individual recent case series have increasingly highlighted—are becoming more prominent in it. A sample of 84 cases alone could not reliably establish whether such a pattern reflects a broader feature of the recent literature or the idiosyncrasies of a small number of publications, a distinction this update is explicitly designed to address (Section 3.2).

The Aim of the Study. In light of the above, this study has two objectives. The first is to build on the systematic review by Bera and Sarkar [5] by updating it to include literature published between September 2014 and August 2026, using the same data-extraction grid to ensure direct comparability with the original sample. The second is to determine whether the expansion of the case series has systematically altered the demographic, diagnostic, clinical, and aetiological profile of the disorder as described in 2015—that is, whether recently published cases differ from earlier reports along any of these dimensions—or whether it has simply confirmed and consolidated its general characteristics.

## 2. Methods

### 2.1. Study Design

This work consists of an update to a previously published systematic review [5], conducted according to a logic of methodological continuity. The study-design eligibility criteria (case reports and case series describing individual cases of delusion of pregnancy in humans) and the data-extraction grid replicate those employed in the original study in order to ensure direct comparability between the historical sample and the one collected here. The search strategy, however, differs from the original review in one respect, described and justified in Section 2.2.

### 2.2. Search Strategy

The literature search was conducted using three databases—Google Scholar, PubMed, and Scopus—with the search last updated in August 2026. Two complementary citation-tracking procedures were also applied and are reported separately, as they involve distinct directions of search. Forward citation searching used Google Scholar’s “Cited by” function, starting from Bera and Sarkar [5] and from each article subsequently included, to identify more recent publications citing these sources. Backward citation searching consisted of a manual review of the reference lists of all included articles to identify any additional eligible studies not captured by the database searches. The time window of interest covered the period following the end of Bera and Sarkar’s search (August 2014) up to August 2026. For each database, search strings were syntactically adapted to the specific Boolean syntax and query fields supported, reported in full in Table 1 together with the exact search date and the filters applied, in order to ensure full reproducibility of the search. Note: AI was used exclusively for formatting the manuscript, preparing the tables (not their content), and checking the English translation. The product was manually reviewed by the authors.

The present search strategy differs from that of Bera and Sarkar [5] in one respect: PsycINFO, one of the three databases used in the original review (alongside PubMed and Google Scholar, both retained here), was replaced with Scopus. This substitution was made because the case-report literature on delusion of pregnancy is published predominantly in general clinical and biomedical journals rather than in psychology-specific outlets; Scopus offers substantially broader coverage of this literature than PsycINFO, whose indexing is focused on psychological and behavioural-science journals, and its integrated citation-tracking functions complement the forward/backward snowballing strategy already applied via Google Scholar. The eligibility criteria, data-extraction grid, and independent dual-reviewer process (screening and extraction conducted separately by both authors, as in the original review) were otherwise unchanged. Embase was considered but not searched; this is acknowledged as a limitation in Section 4.4.

All 134 Google Scholar results were screened in full, in order of relevance ranking (10 results per page); no partial-screening or early-stopping rule was applied, since the query’s total yield was already well below Google Scholar’s approximate 1000-result (≈100-page) display cap, a known platform constraint that did not come into play for this search. The search yielded 134 results on Google Scholar, 61 on PubMed, and 44 on Scopus, for a total of 239 records. After removing 16 duplicates, 223 records were screened by title and abstract. Of these, 202 were excluded at this stage for lack of relevance (absence of individual clinical case descriptions, language other than English, overlap with cases already included in the original 2015 review, or absence of patient-level data), leaving 21 publications subjected to full-text reading for eligibility assessment. The entire selection process, conducted according to the PRISMA 2020 flow diagram [15], is illustrated in Figure 1.

### 2.3. Inclusion and Exclusion Criteria

The inclusion and exclusion criteria replicate those employed by Bera and Sarkar [5]: all case reports and case series describing individual cases of delusion of pregnancy in human beings were included. For a case to be classified as delusion of pregnancy specifically, the source report was required to describe a fixed, false conviction of being pregnant that was either (a) explicitly labelled as delusional or psychotic by the original authors, or (b) independently consistent with the definitional criteria for a delusion—namely, persistence of the belief despite clear contradictory evidence (e.g., a negative pregnancy test, physical examination, or passage of the expected delivery date) and, where reported, impaired reality-testing specific to the pregnancy belief. This criterion is reinforced structurally by the search strategy itself (Section 2.2), whose search strings required the terms “delusion of pregnancy” or “delusional pregnancy” (or close variants) to appear in the title or abstract of retrieved records, so that in practice nearly all candidate records had already been labelled as delusional by their own authors prior to full-text screening. Consistent with the Scopus document-type filter reported in Table 1, this eligibility criterion was applied to peer-reviewed journal articles as well as to conference proceedings and abstract supplements indexed in the databases searched (e.g., European Psychiatry congress abstracts), provided the reported clinical detail was sufficient to extract the variables listed in Section 2.4; purely journalistic or non-indexed grey-literature sources were not considered. Articles concerning pseudocyesis, simulated pregnancy (malingering or factitious), pseudopregnancy, and couvade syndrome were excluded, as these are clinical conditions distinct from delusion of pregnancy proper. As in the original review, eligibility was restricted to articles published in English.

Of the 21 publications subjected to full-text reading, 3 were excluded for the following reasons. One article [16] described a condition explicitly framed by the authors themselves as shared pseudocyesis (folie à deux), without a formalised psychotic/delusional diagnosis for either partner, thus meeting the pseudocyesis exclusion criterion. A second article [17] described a picture of pregnancy-related anxiety with intact reality-testing, diagnosed by the authors themselves as undifferentiated somatoform disorder rather than as a delusion. A third article [18] described a delusion of maternity (the conviction of having already given birth to a child two years earlier), phenomenologically distinct from the delusion of an ongoing pregnancy and more correctly attributable to the spectrum of Delusional Procreation Syndrome [19]. A further borderline distinction, concerning case-level eligibility rather than the exclusion of whole publications, involved three of the seven cases in the series by Sisek-Šprem et al. [20]. One patient described having already given birth to an extremely large number of children while cyclically enacting labour and then reverting to an active belief of being pregnant; a second, while also reporting a long-standing delusional history of having borne several children, explicitly and repeatedly affirmed being currently pregnant at the time of the assessment described in the report; a third believed two children had been born prematurely and placed in an incubator before being taken from her, while simultaneously holding a continuous, unremitting conviction of being pregnant, to the point of declining medication out of concern for the unborn child. In each of these three cases, therefore, the retrospective birth narrative coexisted with an explicit, active belief of an ongoing pregnancy at the time of clinical assessment. Cases were included if they involved such an active, current pregnancy belief, irrespective of whether it coexisted with other delusional content concerning a past pregnancy or birth, and were excluded only when the delusional content was exclusively retrospective, as in Johra and Uddin [18], where no active pregnancy belief was present. All borderline cases were adjudicated jointly by both reviewers under this rule (Section 2.4). The remaining 18 publications, comprising a total of 32 individual cases, were included in the analysis [7,20,21,22,23,24,25,26,27,28,29,30,31,32,33,34,35,36] (Table S1a,b, Supplementary Materials).

### 2.4. Study Selection and Data Extraction

Title and abstract screening, full-text eligibility assessment, and data extraction were all conducted independently and in duplicate by both authors, who participated jointly in every stage of the search, screening, and extraction process; neither stage was delegated to a single reviewer. Disagreements were resolved through discussion between the two reviewers until consensus was reached. A third, independent reviewer was available to arbitrate unresolved disagreements but was not required at any stage of the review. Independent duplicate assessment was considered particularly important given the occasionally ambiguous phenomenological boundary between delusion of pregnancy and related constructs (pseudocyesis, delusion of maternity, and other presentations within the broader spectrum of delusional procreation syndrome), several instances of which required joint discussion before a final eligibility decision was reached (see Section 2.3). The review was guided by a predefined internal protocol, agreed by both authors in advance, that specified the search strategy, eligibility criteria, and data-extraction grid; this protocol was not, however, prospectively registered in a public registry such as PROSPERO, a limitation discussed in Section 4.4.

No formal methodological quality or risk-of-bias appraisal (e.g., the JBI critical appraisal tools for case reports and case series) was applied to the included studies. This choice and its implications for the interpretation of our findings are discussed as a limitation in Section 4.4.

For each case included, the same variables employed in Bera and Sarkar’s [5] original grid were extracted, organised into three areas: (a) demographic characteristics—age, sex, marital status, geographic region of origin; (b) clinical and treatment characteristics—primary psychiatric diagnosis, type of conviction (delusional or ideational), duration of symptom, number of believed foetuses, presence of auditory hallucinations referring to the foetus, subjective perception of foetal movement, treatment response; (c) aetiological factors—psychosocial factors implicated, presence of coenaesthopathic processes, socio-cultural factors, drug-naive status at onset, presence of hyperprolactinaemia. The same categories used in the original study were applied for each variable to allow direct summation.

Several of these categories (e.g., wish-fulfilment, coenaesthopathic processes, socio-cultural factors, psychiatric–medical comorbidity) are interpretive rather than directly reported: they were coded when either (a) the original authors explicitly used an equivalent label or description, or (b) the clinical narrative was judged to be clearly consistent with the operational definition of the category, in the absence of an equally or more consistent alternative. Full operational definitions for each interpretive category, adapted from the categorical structure introduced by Bera and Sarkar [5], are provided in Table S2 (Supplementary Materials) to allow independent audit of these coding decisions. Category assignment was part of the data-extraction process described above and was therefore performed independently and in duplicate by both authors, with disagreements resolved by discussion (Section 2.4).

The complete case-level extraction matrix, reporting every variable listed above for each of the 32 new cases individually, is provided in Table S1a (demographic and clinical/treatment characteristics) and Table S1b (aetiological factors) in the Supplementary Materials so that the classification underlying every aggregated statistic in Table 2, Table 3, Table 4 and Table 5 can be independently audited.

Treatment response was coded into four categories—full, partial, resistant, or not specified—based exclusively on the clinical outcome described in the source publication, irrespective of the specific intervention (s) administered (pharmacological, electroconvulsive, or purely somatic/medical treatment of an underlying organic cause). Cases were coded as full when the source report explicitly described complete resolution or disappearance of the delusional belief (e.g., “delusion resolved”, “full response”, “resolved spontaneously”); as partial when the report described incomplete or partial improvement (e.g., “partial response”, a quantified improvement short of full resolution, or improvement in some but not all delusional content); as resistant when the report explicitly described persistence of the delusion despite treatment or an absence of response; and as not specified when no outcome was reported, or the patient was lost to follow-up before an outcome could be assessed. Because follow-up duration, assessment methods, and criteria for judging improvement varied substantially and were rarely defined explicitly across the included reports, this classification is a descriptive summary of the outcome as reported by the original authors, not a validated or standardised outcome measure, and should not be interpreted as an estimate of true treatment effectiveness. For the same reason, the term “full reported response” is used throughout in preference to “remission”, since no included case report defined or applied formal remission criteria.

### 2.5. Data Synthesis

Data extracted from the 32 new cases were summed, category by category, with the counts published by Bera and Sarkar [5] for the 84 original cases, recalculating percentages on the new combined denominator (*n* = 116). The comparison between the original subsample (*n* = 84) and the new cases (*n* = 32) was conducted on a descriptive basis, reporting absolute counts and percentages separately for each subsample; given the nature of the synthesis (an aggregation of case reports heterogeneous in format and reporting quality), no formal statistical significance tests were applied to the comparison between subsamples, and percentage differences were interpreted qualitatively, with the caveats discussed in Section 4.

To test the robustness of the combined-sample estimates to the borderline case-level eligibility decisions described in Section 2.3, a sensitivity analysis was conducted by recalculating all descriptive statistics after excluding the three Sisek-Šprem et al. [20] cases whose delusional content combined an active pregnancy belief with a co-occurring retrospective birth narrative. Results of this sensitivity analysis are reported in Section 3.2 (Table 6).

A second sensitivity analysis addressed the possibility that the new-case findings are driven by clustering within a small number of publications: key percentages were recalculated after excluding the five cases contributed by the single largest case series [27]. Results are reported in Section 3.2 (Table 7).

This review is reported in accordance with the PRISMA 2020 statement [15]; the completed PRISMA 2020 checklist is provided in Table S3 (Supplementary Materials).

## 3. Results

### 3.1. Combined Sample (N = 116)

The integration of the 32 new cases identified through the systematic search (September 2014–August 2026) with the 84 cases from Bera and Sarkar’s [5] review brings the overall sample to 116 cases.

**Demographic data.** The distribution by age group is reported in Table 2, along with sex, marital status, and geographic region of origin. Female sex accounts for 79.3% of the sample (*n* = 92), and male sex for 20.7% (*n* = 24). The most represented marital status is married (39.7%, *n* = 46). The geographic distribution places Europe first (44.0%, *n* = 51), followed by Asia (39.7%, *n* = 46).

**Clinical data.** The most frequent primary psychiatric diagnosis is schizophrenia (35.3%, *n* = 41), followed by bipolar disorder (12.1%, *n* = 14) and psychiatric–medical comorbidity (12.1%, *n* = 14). The conviction is of the delusional type in 98.3% of cases (*n* = 114). Symptom duration is less than 3 months in 34.5% of cases (*n* = 40) and between 3 months and 1 year in 24.1% (*n* = 28). The delusion concerns a single foetus in 78.4% of cases (*n* = 91). Subjective perception of foetal movement is reported in 45.7% of cases (*n* = 53); auditory hallucinations specifically referring to the foetus are present in 9.5% (*n* = 11). Treatment response is reported as full in 60.3% of cases (*n* = 70) and partial in 13.8% (*n* = 16). Details for each variable are reported in Table 3.

**Aetiological factors.** Among psychosocial factors, the most frequently implicated is wish-fulfilment (26.7%, *n* = 31); in 31.0% of cases (*n* = 36), no specific psychosocial factor is implicated. Coenaesthopathic processes are documented in 26.7% of cases (*n* = 31). Socio-cultural factors are not implicated in 63.8% of cases (*n* = 74). Drug-naive status at onset is documented in 32.8% of cases (*n* = 38). Hyperprolactinaemia is implicated in 20.7% of cases (*n* = 24). Details are reported in Table 4.

### 3.2. Comparison Between the Original Subsample (N = 84) and New Cases (N = 32)

Table 5 reports the direct comparison, for the variables with the greatest percentage gap between the two subsamples, between the percentages of Bera and Sarkar’s original sample and those calculated on the 32 new cases identified in this update.

The variables with the greatest percentage gap between the two subsamples are: organic disorder diagnosis, age over 60 years, coenaesthopathic processes, hyperprolactinaemia, and unspecified data for auditory hallucinations, drug-naive status, and perception of foetal movement. The remaining variables (sex, marital status, type of conviction, number of believed foetuses, treatment response) show percentage gaps contained within 15 percentage points between the two subsamples.

As a sensitivity check on the case-level eligibility decisions discussed in Section 2.3, all combined-sample statistics were recalculated after excluding the three Sisek-Šprem et al. [20] cases whose delusional content combined an active pregnancy belief with a co-occurring retrospective birth narrative (sensitivity sample: *n* = 113). As shown in Table 6, the five headline percentages most discussed in this manuscript changed minimally under this stricter case definition, with a maximum absolute change of 2.1 percentage points (hyperprolactinaemia); no qualitative conclusion reported in this update would differ under the sensitivity analysis.

A second sensitivity analysis addressed publication-level clustering among the new cases: fifteen of the 32 new cases originate from only three publications (seven from Sisek-Šprem et al. [20]; five from Guilfoyle et al. [27]; three from Sharma et al. [7]), raising the possibility that headline differences between the original and updated subsamples reflect the characteristics of a small number of case series rather than a broader pattern independently replicated across the new literature. This is particularly relevant for organic diagnosis and coenaesthopathic processes, the two variables showing the largest historical/updated differences (Section 3.2, Table 5), given that Guilfoyle et al. [27] contributed five cases specifically selected for an organic, constipation-related presentation. Table 7 recalculates the new-case and combined-sample percentages for these variables, plus age over 60 years, after excluding the five Guilfoyle et al. [27] cases.

A further concern is that missing information in case reports cannot be assumed to represent a true absence of the corresponding clinical feature: an original author’s failure to mention a finding is not equivalent to that finding being absent. For the three variables with the highest proportion of “not specified” data among the new cases—auditory hallucinations, perceived foetal movement, and drug-naive status—Table 8 therefore reports both the conventional total-sample percentage and an available-case percentage restricted to cases for which the relevant information was actually reported. This recalculation is not possible for hyperprolactinaemia, since that variable was coded as a strict binary (yes/no) without a “not specified” category (Section 4.4); we return to this coding asymmetry below. Restricting the denominator to available cases substantially changes the magnitude, and in one instance the apparent direction, of the period comparison: perceived foetal movement rises from 52.8% to 93.8% of available-case reports between the original and new subsamples—a far larger shift than the 45.2% to 46.9% suggested by the total-sample percentages alone—while auditory hallucinations and drug-naive status show a comparable pattern of amplification once non-reporting cases are excluded. Because reporting of these variables may itself be non-random (e.g., clinicians may be more likely to document foetal-movement sensations when they are present and clinically salient), neither denominator should be treated as definitive; we present both to make this source of uncertainty transparent rather than to endorse either as the more accurate estimate.

## 4. Discussion

This study had two objectives. The first was descriptive: to update the case series on delusion of pregnancy published by Bera and Sarkar [5], which had compiled 84 cases reported through August 2014. The second objective was to determine whether the eleven subsequent years of literature had substantially altered the clinical and aetiological profile of the disorder or whether they had merely increased the number of reported cases without changing the underlying structure.

Regarding the first objective, the data show that the sample of 116 cases tends to confirm the phenomenological profile already outlined in the previous literature. Schizophrenia remains by far the most common psychiatric diagnosis (35.3%), confirming the findings of Bera and Sarkar [5] and subsequent observations [23,31], which emphasise the centrality of schizophrenia as the most frequent underlying diagnosis. This pattern is consistent across both the original and updated samples; however, the fact that schizophrenia is the most frequently reported diagnosis among published cases cannot, on its own, establish a shared pathogenetic mechanism between the two conditions, and no such claim is intended here. Furthermore, auditory hallucinations—a specific and pivotal symptom of schizophrenia—can fuel delusional content, as in cases where patients report hearing the voices of the foetuses [21]. The predominance of female patients (79.3%) and monofetal presentation (78.4%) also remains essentially stable compared with historical data, as does the good overall reported response to treatment (60.3% full reported response). What the combined sample highlights more clearly than the 2015 cases alone, however, is the aetiological heterogeneity of the phenomenon. The combined proportion of organic cases and cases with psychiatric–medical comorbidities is 23.3% (*n* = 27), a proportion that is by no means negligible and that, in itself, justifies the emphasis—already present in earlier literature but numerically reinforced here—on the need for a comprehensive medical workup before attributing the delusion to an exclusively psychiatric process. Gogia and colleagues [14], in their systematic review of 140 cases of delusion of pregnancy specifically focused on medical and surgical comorbidities, had already identified an associated organic condition in 23 out of 140 cases (gallstones, abdominal masses, tubal cysts), with symptom resolution in 10 out of 15 cases that underwent targeted medical intervention: a finding that this update only serves to confirm and extend.

To assess the completeness of the present update against this frequently cited figure, we cross-referenced the case list underlying Gogia et al.’s 140 cases against the present database. Importantly, the figure of 140 refers to the total yield of their initial, deliberately broader search, which—unlike the present review and Bera and Sarkar’s original review—also used the search terms “pseudocyesis” and “false/pseudo/phantom/spurious pregnancy”; after applying their own inclusion criterion (evidence of a contributing medical or surgical condition), only 23 of these 140 cases were retained in their analytic sample and individually reported. Cross-referencing these 23 cases against the present database shows that 18 were published before September 2014 and therefore fall within the scope of Bera and Sarkar’s original review rather than the present update. Of the remaining five cases published after September 2014, all five—the triplet-pregnancy case reported by Goyal et al. [26] and four of the five cases in the Guilfoyle et al. [27] case series—are already included in the present update (Table S1a,b, Supplementary Materials). Two further post-2014 cases identified by Gogia et al. [37,38] were not captured by our search; both were published in Reactions Weekly, a pharmacovigilance digest rather than a primary peer-reviewed case report, which may explain their absence from the databases searched here. One further case [39] could not be unambiguously dated relative to our September 2014 cut-off, and its inclusion status therefore remains uncertain. Taken together, this reconciliation indicates that the numerical discrepancy between the 140 cases cited from Gogia et al. and the present combined sample of 116 cases is attributable primarily to differences in case definition—specifically, the inclusion of pseudocyesis-related search terms in the earlier review—rather than to eligible delusion-of-pregnancy cases missed by the present search.

The widest discrepancy between the two subsamples concerns organic diagnosis (4.8% vs. 28.1%) and, similarly, the age group over 60 years (17.9% vs. 28.1%). Before these figures are interpreted as evidence of a temporal shift, however, an important caveat must be addressed: the 32 new cases do not constitute 32 independent observations. Fifteen of them originate from only three publications—seven from Sisek-Šprem et al. [20], five from Guilfoyle et al. [27], and three from Sharma et al. [7]—and the five cases contributed by Guilfoyle et al. alone account for a disproportionate share of the organic and geriatric presentations in this update. When these five cases are excluded, the organic-diagnosis proportion among the remaining 27 new cases falls from 28.1% to 14.8%, and the proportion aged over 60 years falls from 28.1% to 14.8%—the latter now below, rather than above, the original 17.9% (Table 7). This indicates that the apparent shift toward organic and geriatric presentations is driven substantially by clustering within a single case series rather than by a broader pattern independently replicated across the new literature, and the finding should therefore be read as a hypothesis worth further investigation rather than as evidence of a population-level temporal change. This does not mean the phenomenon is spurious: Guilfoyle et al. [27] situate their own series within a small pre-existing literature, citing Harland and Warner [40]—five elderly patients, most with documented constipation—and Curran and Pugh [41], in which two of three patients had dementia; and Mehan et al. [30] separately note that only 17.9% of Bera and Sarkar’s original cases involved patients over 60, describing their own case (a 64-year-old man) as a “relative rarity”. Taken together, these sources confirm that geriatric and organic presentations of DoP are a recognised, if still uncommon, phenomenon, but the present update cannot determine whether their apparent increase in frequency reflects a genuine change over time, increased clinical or diagnostic attention, growing awareness prompted by a small number of influential case series, or some combination of these; disentangling these possibilities would require a systematic review explicitly designed to account for clustering at the publication level, which is beyond the descriptive scope of the present update.

Coenaesthopathic processes were reported at a higher proportion among recently published cases than in the original sample (43.8% vs. 20.2%); as with the organic/geriatric finding just discussed, this figure is sensitive to the same clustering. Excluding the five Guilfoyle et al. [27] cases, the proportion among the remaining 27 new cases falls to 33.3% (Table 7)—still elevated relative to the original review, but appreciably less pronounced, and subject to the same caveat regarding population-level inference discussed above. With this caveat in mind, most coenaesthopathic cases in the recent subsample arise from Guilfoyle et al. and from other organic/geriatric cases, where the predominant pathogenic mechanism is not the complex delusional construct typical of schizophrenia, but rather the misinterpretation of somatic signals (abdominal distension, pain, perception of bowel movements) in a context of cognitive decline or acute confusion. Levy et al. [42] had already proposed a two-factor model to explain this mechanism: a “first factor” consisting of abnormal sensory input (for example, hyperprolactinaemia or, in geriatric patients, distension due to faecal impaction) and a “second factor”—a cognitive process—that determines its delusional interpretation. It is possible that, in organic/geriatric cases, this second factor takes on a qualitatively different form, closer to confusional confabulation than to a systematised delusional construct; however, this remains a hypothesis that requires dedicated psychopathological verification in a design not vulnerable to publication clustering, which cannot be derived solely from the frequency analysis conducted here.

The proportion of cases with documented hyperprolactinaemia rose from 16.7% to 31.2% in the recent subsample. Unlike the two previous points, for this variable a purely methodological interpretation appears just as plausible as a clinical one. Prolactin testing is probably more systematic in current clinical practice than it was fifteen years ago, supported in part by a more established body of literature on the role of second-generation antipsychotics in inducing hyperprolactinaemia. Ahuja et al. [43], whose work is cited by nearly all the case reports collected here, had already observed the resolution of the delusion coinciding with the normalisation of prolactin levels in a significant proportion of their patients—an observation that more recent cases [26,29] confirm, although they do not allow us to determine whether the observed increase in frequency reflects a genuine rise in the phenomenon or an improvement in the descriptive standards of recent publications.

### 4.1. Classification and Assessment: A Proposal for an Operational Distinction

The data discussed in the preceding paragraphs suggest, on an exploratory basis, the usefulness of an operational distinction between a form that we might define as primary (of complex delusional origin, typically associated with schizophrenia or another chronic psychotic disorder, with an elaborate thematic structure and frequent coexistence with other persecutory or referential delusions) and a form secondary to somatic misinterpretation (of organic or confusional origin, typically in elderly patients with dementia or delirium, with acute onset, a shorter course, and resolution closely linked to treatment of the underlying somatic cause). It is important to emphasise that this distinction has not yet been formalised in current nosography and should be understood as a hypothesis-generating concept, derived retrospectively from a descriptive pattern observed by association between two heterogeneous subsamples of aggregated case reports, rather than from a study design suitable for establishing causal links. The co-occurrence of organic/geriatric aetiology, a brief course, and coenaesthopathic processes—though common in the cases collected here—does not necessarily imply a single pathogenic mechanism, nor does this distinction currently have demonstrated prognostic validity; its confirmation requires prospective, controlled study designs. This proposal in no way intends to replace the DSM-5 classification, which categorises delusion of pregnancy as a somatic subtype of delusional disorder or as a symptom within the schizophrenia spectrum; rather, it suggests supplementing this classification with a hypothetical pathogenetic criterion. At this stage, however, this criterion remains a research hypothesis rather than a basis for clinical decision-making and should not be applied in clinical practice until it has undergone the prospective, controlled validation described above. A further complication for this proposal is that not all cases fit cleanly into either pole. The combined sample includes 14 cases (12.1%) classified as psychiatric–medical comorbidity, in which a chronic psychotic disorder (typically schizophrenia) co-occurs with a documented organic contributor (e.g., epilepsy, tuberous sclerosis) to the delusional presentation—combining features more typical of the primary and of the secondary form as described above. Such cases suggest that the primary/secondary distinction may be better conceptualised as two poles of a continuum or as two dimensions that can co-occur within the same patient, rather than as a strict binary classification. We have not attempted to formally assign individual cases to either category, nor have we tested the reliability of any such assignment; doing so, and explicitly accommodating mixed or overlapping presentations, is a prerequisite for the prospective validation studies proposed in Section 4.3, not a task undertaken in the present descriptive update.

Independently of whether the primary/secondary distinction is eventually validated, its underlying rationale nonetheless converges with a more general and already well-established clinical principle: organic causes should be systematically ruled out before a psychiatric diagnosis is finalised in patients presenting with delusion of pregnancy. In terms of assessment, this suggests a two-phase evaluation process, offered here as a reasonable clinical approach consistent with general good practice rather than as a validated protocol derived from the proposed classification. In the first phase, regardless of the patient’s age, the organic causes most frequently implicated in the literature reviewed here should be systematically ruled out: a targeted abdominal physical examination to exclude faecal impaction or distension (particularly in elderly or cognitively impaired patients), measurement of prolactin levels with a review of current psychopharmacological therapy, and—when clinically indicated by the patient’s presentation—thyroid and electrolyte screening, given the prevalence of hypothyroidism and electrolyte/glycaemic imbalances among the reported comorbidities [21,24]. Only in the second phase, after the exclusion or treatment of immediately reversible organic causes, should the definitive psychiatric diagnosis be established. A structured data-collection tool could also be proposed as a minimum standard for future case reports, modelled on what the CARE guidelines have already established for clinical case reporting in general; this would make future updates to this case series significantly more comparable than has been possible here for variables with a higher rate of missing data.

### 4.2. Practical Implications

From a pharmacological standpoint, three types of statement should be kept distinct. A purely descriptive observation from the present synthesis is that, in cases with an organic or coenaesthopathic aetiology, treatment of the underlying somatic cause (e.g., correcting constipation, electrolyte imbalances, or thyroid dysfunction) was followed by resolution of the delusion without escalation of antipsychotic therapy in several of the cases reviewed here; this describes what was reported in these specific cases, not a demonstrated comparative treatment effect, since no comparison group or controlled design was available. Separately, an established recommendation from the independent pharmacological literature, applicable to antipsychotic-induced hyperprolactinaemia in general rather than derived from this case series, favours prolactin-sparing agents (e.g., aripiprazole, quetiapine) on pharmacological grounds when hyperprolactinaemia is implicated. Whether preferential use of such agents specifically improves outcomes in DoP patients with hyperprolactinaemia-associated delusions, relative to other antipsychotics, has not to our knowledge been directly tested and remains a hypothesis requiring prospective, comparative study.

From a psychotherapeutic perspective, the same three-way distinction applies. Two purely descriptive, single-case observations are reported in the literature reviewed here, neither constituting evidence of comparative efficacy: Mehan et al. [30] describe a guided reality-testing approach in which the patient consented to take a pregnancy test as concrete evidence contradicting the delusional belief, introduced gradually and non-confrontationally; and Arora et al. [22] describe supportive psychodynamic psychotherapy exploring underlying themes (e.g., fear of pregnancy within a problematic relationship, desire for a son) alongside pharmacological treatment, with reduction in delusional belief reported within two weeks in a single patient. Separately, and drawing on the independent, controlled literature on delusional symptoms more broadly rather than on DoP specifically, reality-testing following a verbal challenge to the delusional belief has been reported as among the more effective interventions for modifying delusional ideas [44], and a recent single-blinded randomised-controlled trial supports an emotion-oriented cognitive-behavioural approach that targets the mechanisms maintaining delusions rather than their content directly [45]. Whether these approaches, established for delusional symptoms in general, generalise specifically to DoP—and whether the two single-case interventions described above would replicate in a larger, controlled sample—remains a hypothesis requiring prospective testing rather than a demonstrated finding. Family psychoeducation is a reasonable adjunct to consider in family contexts that appear to act as a stressor [31,34], though this too reflects a descriptive observation from individual cases rather than a tested intervention.

### 4.3. Future Directions

The body of data discussed highlights several areas that future research should explore through empirical investigation—areas that a simple review of clinical cases certainly cannot address. The first area, and likely the most urgent, concerns the empirical validation of the primary/secondary distinction that appears to emerge from the case analysis. Future studies could, for example, conduct multicentre cohort studies that prospectively enrol patients with delusion of pregnancy, stratifying them by suspected aetiology (organic/geriatric vs. primary psychotic) from the outset of care, and/or systematically compare course, response to treatment, and phenomenological characteristics between cases with probable organic/geriatric aetiology and cases with probable primary psychotic aetiology, including a comparison group (e.g., patients with delirium or dementia without delusion of pregnancy) in order to isolate the truly distinctive factors.

The second area involves a more in-depth examination of the role of adverse developmental experiences and collective traumatic events or acute social deprivation as aetiological risk factors in an attempt to move beyond or supplement the more traditional concept of individual wish-fulfilment that dominates the classical psychodynamic interpretation of symptoms. The case reported by Tezcan and Turan [34], whose patient lived in a container settlement following the 2023 Turkish earthquake, and that of Tascon Guerra et al. [33], whose patient developed delusions after months of isolation during the COVID-19 quarantine, suggest an area of investigation that has not yet been systematically explored. A study of clinical populations exposed to natural disasters, conflicts, or prolonged social isolation could therefore clarify whether this pattern represents a genuine causal association or a reporting artefact linked to the temporal overlap between events of public significance and the publication of case reports.

The third area concerns people with intellectual disabilities. Delusion of pregnancy appears to be systematically underdiagnosed and underreported, owing both to the scarcity of validated psychopathological assessment tools for this population and to the difficulty of distinguishing content-poor delusional thinking from developmentally expected behaviours. The prospective development and validation of screening tools specific to psychotic symptoms in patients with intellectual disabilities would make it possible to determine whether the current scarcity of reported cases in this population reflects a genuinely lower incidence or, as suggested by Ursumando et al. [35] themselves, a systematically under-recognised phenomenon.

A fourth area, of a more strictly medico-legal nature, concerns two under-explored dimensions of forensic risk. The case reports compiled here document isolated instances of behaviour with potential forensic relevance—self-harm associated with attempts to verify the delusional belief [31] and accusatory behaviour directed at healthcare staff [34]—but this evidentiary base (two cases) is far too limited to estimate risk or to inform practice recommendations. We caution explicitly against extrapolating findings from the denial-of-pregnancy and postpartum-psychosis literature, where an association with neonaticide has been documented [10], to delusion of pregnancy: these are phenomenologically distinct conditions, and no evidence collected here or elsewhere supports an analogous risk of violence in DoP. Separately, the literature compiled here contains no cases documenting the possible impact of delusion of pregnancy on mental capacity during criminal proceedings. Future studies, including reviews of forensic case series and systematic collection of the behavioural observations noted above, could examine whether, and under what circumstances, an active delusion of pregnancy at the time of an offence has led to a finding of total or partial insanity, and whether the primary/secondary distinction proposed in Section 4.1—itself still unvalidated—is reflected in the expert evaluations actually conducted.

### 4.4. Limitations

This update shares the inherent limitations of any synthesis based on isolated case reports: the absence of a control group, the inability to estimate the true incidence of the phenomenon, and a likely publication bias favouring atypical clinical presentations, which could explain, at least in part, the increase in the proportion of organic/geriatric cases observed here. In addition, there is heterogeneity in reporting between the two subsamples, which calls for caution in interpreting the “data not specified” percentages as an actual absence of the phenomenon in the more recent subsample, where these percentages are systematically higher. More broadly, clinical and diagnostic practices, including routine laboratory workup, psychiatric nosology, and case-reporting conventions, have themselves changed over the eleven-year span separating the two subsamples; any comparison between them therefore reflects both possible changes in the disorder’s presentation and possible changes in how it is investigated and reported, which cannot be disentangled with the present design. Finally, the literature search for this update prioritised English-language publications, thereby replicating the same linguistic limitation already noted by the authors of the original review. This restriction should be understood as a substantive source of language bias, not merely a technical limitation: for a condition as rare as DoP, individual case reports from non-English-speaking geographical and cultural settings may be disproportionately excluded, and any cross-cultural variation in symptom presentation, aetiological attribution, or reporting practice in those settings cannot be assessed from the present sample. Relatedly, Embase was not searched; while Scopus and PubMed provide substantial overlapping coverage of the biomedical literature relevant to this topic, Embase’s distinct indexing and its particular strength in pharmacological and drug-safety literature mean that some eligible reports—for example, those focused primarily on medication-induced hyperprolactinaemia or other pharmacologically mediated presentations—may not have been captured by the present search. A further limitation is that, although the review followed a predefined internal protocol with independent, duplicate screening and extraction (Section 2.4), this protocol was not prospectively registered in a public registry such as PROSPERO; formal registration was not undertaken, and its absence should be considered when weighing the evidentiary strength of this update relative to prospectively registered systematic reviews.

A further limitation concerns the absence of a formal methodological quality or risk-of-bias appraisal of the included case reports and case series (e.g., using the JBI critical appraisal checklists for case reports and case series). This appraisal was not performed, for three main reasons. First, the heterogeneity of the included literature—spanning full case reports, brief case series, and short communications or conference abstracts—makes the uniform application of standardised critical appraisal criteria difficult, since several of the shorter formats do not report the methodological detail (e.g., systematic follow-up, explicit diagnostic confirmation, structured assessment of adverse events) that such tools require in order to be scored meaningfully. Second, the original review by Bera and Sarkar [5], whose methodology this update was designed to replicate for direct comparability, likewise did not include a formal quality appraisal; introducing one only for the newly identified cases would have created an asymmetry between the historical and updated subsamples that could itself have biased the comparison presented in Section 3.2. Third, and most importantly, the absence of quality appraisal means that all descriptive frequencies reported in this review—including treatment response, aetiological factors, and the primary/secondary distinction proposed in Section 4.1—should be interpreted as unweighted summaries of heterogeneous evidence of variable reliability, rather than as quality-adjusted estimates. In practice, this means that a detailed case report with comprehensive diagnostic assessment contributes to the aggregated counts in exactly the same manner as a case with substantial missing information; no weighting scheme was applied to differentiate them. This consideration reinforces, rather than undermines, the explicitly exploratory and hypothesis-generating framing already given to the primary/secondary distinction (Section 4.1) and underscores the need for future studies validating that distinction to incorporate formal risk-of-bias assessment as part of their design.

A related and more fundamental limitation concerns the handling of missing information. For some variables (e.g., perceived foetal movement, auditory hallucinations, duration, drug-naive status), an explicit “not specified” category was retained, following the coding scheme of Bera and Sarkar [5], to distinguish cases in which the relevant information was genuinely absent from the source report. For other variables, however, the same three-way distinction was not applied, and cases were instead coded exclusively as “yes” or “no” (hyperprolactinaemia) or as the corresponding factor “not implicated” (coenaesthopathic processes, psychosocial factors, socio-cultural factors). This inconsistency reflects, in part, a genuine difference in the nature of these variables. Hyperprolactinaemia is an objective laboratory finding, and its absence from a case report cannot automatically be equated with a normal prolactin value, since many case reports—particularly older ones—did not report prolactin testing at all. Coenaesthopathic processes and psychosocial or socio-cultural factors, by contrast, are interpretive categories inferred by reviewers from the clinical narrative rather than directly reported findings, for which a judgement of “not implicated” already reflects an absence of supporting textual evidence in the source report rather than a claim about the patient’s true underlying state. In both cases, however, the “no”/”not implicated” counts reported in Table 4 and Table 5 should be interpreted as a lower bound on the true prevalence of these factors, since some proportion of these cases may reflect non-assessment or non-reporting by the original authors rather than confirmed absence. This limitation could not be corrected retrospectively for the 84 cases from Bera and Sarkar [5], since only their aggregated, already-classified frequencies were available for this update, not the underlying individual-case data; for consistency and direct comparability, the same binary coding convention was therefore also applied to the 32 new cases, even though the reviewers had access to the original full texts and could, in principle, have applied a more granular three-way classification (reported present; reported absent; not assessed/not reported) to this subset alone. Future updates of this case series may wish to adopt such a three-way classification prospectively for all variables, which would also make it possible to reassess whether the historical Bera and Sarkar [5] counts can be usefully supplemented with this additional granularity.

Finally, and more generally, this evidence base—a descriptive synthesis of selectively published case reports and case series, without a comparison group or prospective sampling frame—cannot establish the true population prevalence or incidence of delusion of pregnancy, nor can it support causal or pathogenetic conclusions about any of the clinical, aetiological, or diagnostic associations discussed in this review, including the primary/secondary distinction proposed in Section 4.1. All such associations should be read as hypotheses generated by, rather than established by, the present update.

## 5. Conclusions

This update integrates 32 newly identified cases with Bera and Sarkar’s [5] original 84, forming a combined descriptive sample of 116 published cases of delusion of pregnancy. Three conclusions can reasonably be drawn from this evidence. First, the general phenomenological profile described in 2015—female predominance, schizophrenia as the most frequently reported underlying diagnosis, monofetal presentation, and a generally favourable reported treatment response—is substantially confirmed in the enlarged sample. Second, the higher proportion of organic/geriatric presentations and coenaesthopathic processes among recently published cases is real at the level of the published record, but sensitivity analysis indicates that it is substantially attenuated once publication-level clustering is accounted for (Section 3.2, Table 7); it should therefore be read as a pattern in the literature reviewed, not as evidence of a population-level temporal or epidemiological change. Third, the proposed distinction between a primary, delusional form and a secondary, organically driven form of DoP (Section 4.1) remains, at this stage, a hypothesis generated from these descriptive data rather than a validated clinical classification; it is offered as a direction for future prospective, controlled research and should not presently be used to guide individual clinical decisions. Beyond these points, the comprehensive case-level extraction matrix provided in the Supplementary Materials is intended as a resource for future updates and independent re-analysis of this literature.

## Figures and Tables

**Figure 1:**
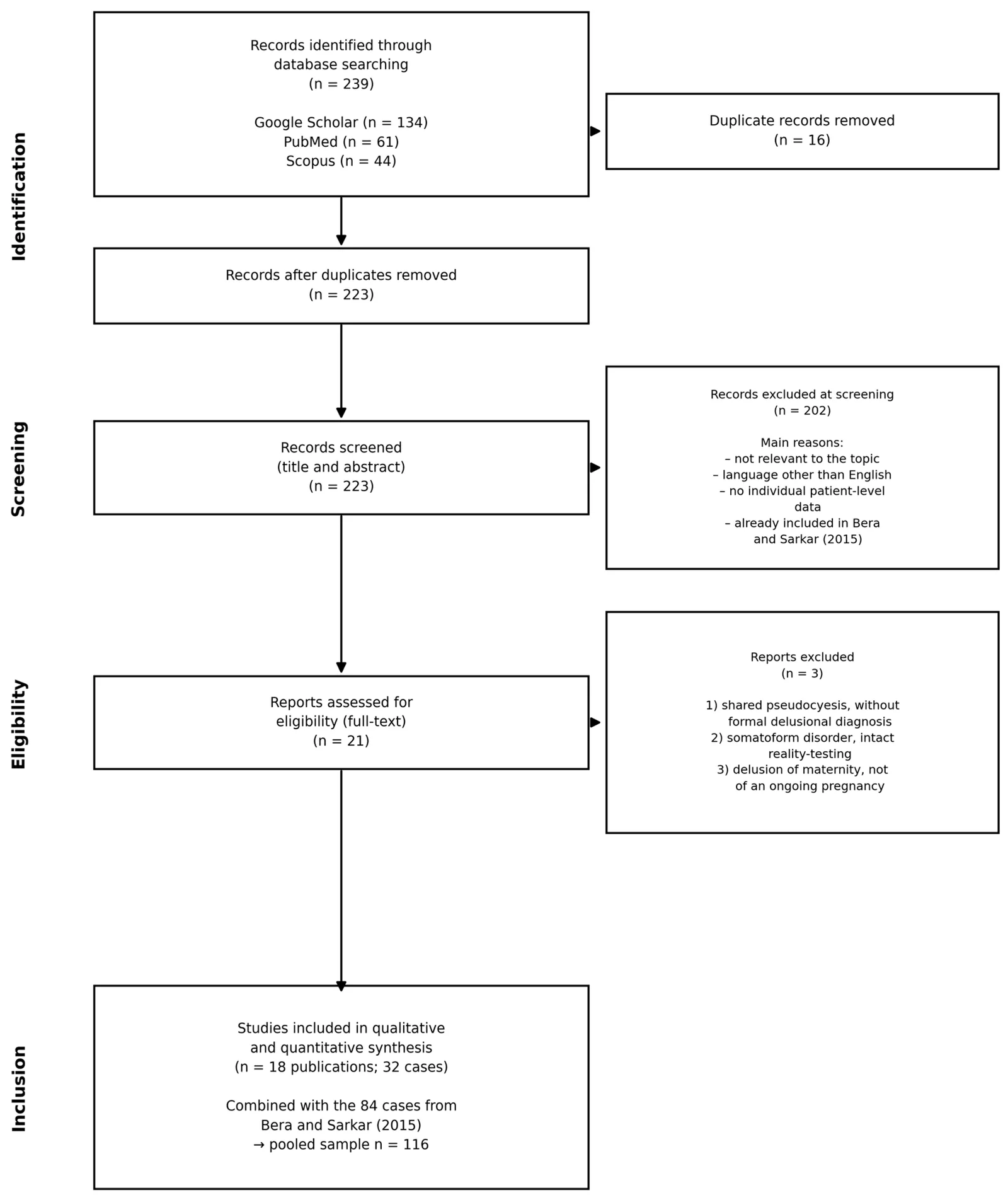
PRISMA flow diagram of study selection. *Note.* The diagram follows the PRISMA 2020 Statement [15]. The reasons for exclusion reported in the “Reports excluded” box are described in detail in Section 2.3. Bera and Sarkar [5].

**Table 1:** Search strings, filters applied, and search date for each database.

Database	Search Date	Search String	Filters Applied	Records Retrieved
Google Scholar	3 August 2026	“delusion of pregnancy” OR “delusional pregnancy” OR “false pregnancy delusion” OR “pregnancy delusion”	Custom date range 2014–2026; patents and citations without full-text excluded; “Cited by” function applied for forward citation searching, starting from Bera and Sarkar [5] and from each article subsequently included; reference lists of all included articles were separately reviewed for backward citation searching	134
PubMed	4 August 2026	(“delusion *” [Title/Abstract] AND “pregnant *” [Title/Abstract]) OR “delusion of pregnancy” [Title/Abstract] OR “delusional pregnancy” [Title/Abstract] OR “false pregnancy delusion” [Title/Abstract] OR “pregnancy delusion” [Title/Abstract]	Publication date: 1 September 2014–31 August 2026; Species: Humans; Language: English	61
Scopus	5 August 2026	TITLE-ABS-KEY ((delusion * AND pregnant *) OR “delusion of pregnancy” OR “delusional pregnancy” OR “false pregnancy delusion” OR “pregnancy delusion”) AND PUBYEAR > 2013 AND LIMIT-TO (LANGUAGE, “English”) AND (LIMIT-TO (DOCTYPE, “ar”) OR LIMIT-TO (DOCTYPE, “re”) OR LIMIT-TO (DOCTYPE, “cp”))	PUBYEAR > 2013, with manual verification of publication month to exclude records predating September 2014; Language: English; Document type: article, review, conference paper	44

*Note.* The Google Scholar search strings use the simple syntax supported by the engine (no advanced Boolean fields); the PubMed and Scopus strings use the [Title/Abstract] field tag and the TITLE-ABS-KEY field, respectively, with their associated truncation operators (*) and LIMIT-TO filters.

**Table 2:** Demographic characteristics of the combined sample (*n* = 116).

Variable	*n*	%
*Age (years)*		
≤20	8	6.9%
21–30	24	20.7%
31–40	23	19.8%
41–50	24	20.7%
51–60	13	11.2%
>60	24	20.7%
*Sex*		
Female	92	79.3%
Male	24	20.7%
*Marital status*		
Married	46	39.7%
Unmarried	37	31.9%
Separated/divorced/widowed	21	18.1%
Not specified	12	10.3%
*Geographic region*		
Europe	51	44.0%
Asia	46	39.7%
Americas	9	7.8%
Africa	8	6.9%
Oceania	1	0.9%
Not specified	1	0.9%

**Table 3:** Clinical and treatment characteristics of the combined sample (*n* = 116).

Variable	*n*	%
*Primary diagnosis*		
Schizophrenia	41	35.3%
Bipolar disorder	14	12.1%
Psychiatric–medical comorbidity	14	12.1%
Organic disorder	13	11.2%
Depressive disorder	10	8.6%
Other	10	8.6%
Other psychotic disorder	9	7.8%
Schizoaffective disorder	4	3.4%
Neurotic disorder	1	0.9%
*Type of conviction*		
Delusional	114	98.3%
Ideational	2	1.7%
*Duration of symptom*		
<3 months	40	34.5%
3 months to 1 year	28	24.1%
1–2 years	5	4.3%
2–5 years	5	4.3%
5–10 years	3	2.6%
>10 years	7	6.0%
Not specified	28	24.1%
*Number of believed foetuses*		
Single	91	78.4%
Twin	7	6.0%
Triplet	8	6.9%
Quadruplet or more	10	8.6%
*Perceived foetal movement*		
Yes	53	45.7%
No	35	30.2%
Not specified	28	24.1%
*Auditory hallucinations (foetus)*		
Yes	11	9.5%
No	84	72.4%
Not specified	21	18.1%
*Treatment response*		
Full	70	60.3%
Partial	16	13.8%
Resistant	8	6.9%
Not specified	22	19.0%

**Table 4:** Aetiological factors of the combined sample (*n* = 116).

Variable	*n*	%
*Psychosocial factors*		
Unfulfilled desire (wish-fulfilment)	31	26.7%
Other	16	13.8%
Loss of a loved object/relationship	10	8.6%
Rebirth fantasies	6	5.2%
Disturbed family dynamics	4	3.4%
Sexual abuse/sexual identity confusion	4	3.4%
Over-identification/rivalry/projection	4	3.4%
Strong identification with pregnant women	3	2.6%
Parental deprivation	1	0.9%
Intense ambivalence toward pregnancy	1	0.9%
Not implicated	36	31.0%
*Coenaesthopathic processes*		
Yes	31	26.7%
No	85	73.3%
*Socio-cultural factors*		
Not implicated	74	63.8%
Excessive societal pressure to procreate	15	12.9%
Other	12	10.3%
Chronic social deprivation	7	6.0%
Supernatural beliefs/evil eye	8	6.9%
*Drug-naive status at onset*		
Yes	38	32.8%
No	56	48.3%
Not specified	22	19.0%
*Hyperprolactinaemia*		
Yes	24	20.7%
No	92	79.3%

**Table 5:** Comparison between the original sample (*n* = 84), the new cases (*n* = 32), and the combined sample (*n* = 116).

Variable	Bera & Sarkar [5] (*n* = 84)	New Cases (*n* = 32)	Combined (*n* = 116)
**Age >60 years**	15 (17.9%)	9 (28.1%)	24 (20.7%)
**Organic diagnosis**	4 (4.8%)	9 (28.1%)	13 (11.2%)
**Coenaesthopathic processes (Yes)**	17 (20.2%)	14 (43.8%)	31 (26.7%)
**Hyperprolactinaemia (Yes)**	14 (16.7%)	10 (31.2%)	24 (20.7%)
**Auditory hallucinations (not specified)**	6 (7.1%)	15 (46.9%)	21 (18.1%)
**Drug-naive status (not specified)**	6 (7.1%)	16 (50.0%)	22 (19.0%)
**Foetal movement (not specified)**	12 (14.3%)	16 (50.0%)	28 (24.1%)

*Note.* Only the variables with the widest percentage gap between Bera and Sarkar’s [5] original sample and the new cases identified in this update are reported.

**Table 6:** Sensitivity analysis: combined-sample percentages with and without the three borderline Sisek-Šprem et al. [20] cases.

Variable	Full Sample (*n* = 116)	Sensitivity Sample, Excl. B/E/F (*n* = 113)	Δ (pp)
Age >60 years	24 (20.7%)	24 (21.2%)	+0.5
Organic diagnosis	13 (11.2%)	12 (10.6%)	−0.6
Coenaesthopathic processes (Yes)	31 (26.7%)	31 (27.4%)	+0.7
Hyperprolactinaemia (Yes)	24 (20.7%)	21 (18.6%)	−2.1
Treatment response (Full)	70 (60.3%)	70 (61.9%)	+1.6

*Note*. Δ = percentage-point change from the full combined sample (*n* = 116) to the sensitivity sample excluding cases B, E, and F from Sisek-Šprem et al. [20] (*n* = 113).

**Table 7:** Sensitivity analysis for publication-level clustering: key percentages with and without the five cases contributed by Guilfoyle et al. [27].

Variable	New Cases, Full (*n* = 32)	New Cases, Excl. Guilfoyle et al. (*n* = 27)	Combined, Full (*n* = 116)	Combined, Excl. Guilfoyle et al. (*n* = 111)
Organic diagnosis	9 (28.1%)	4 (14.8%)	13 (11.2%)	8 (7.2%)
Coenaesthopathic processes (Yes)	14 (43.8%)	9 (33.3%)	31 (26.7%)	26 (23.4%)
Age >60 years	9 (28.1%)	4 (14.8%)	24 (20.7%)	19 (17.1%)

*Note*. “Full” columns reproduce the figures already reported in Table 2, Table 3, Table 4 and Table 5; “excl. Guilfoyle et al.” columns recalculate the same percentages after removing the five cases contributed by that case series (new-case *n* = 27; combined *n* = 111). Age > 60 years falls below, rather than above, the original Bera and Sarkar [5] proportion (17.9%) once these cases are excluded.

**Table 8:** Total-sample versus available-case percentages for the three variables with the highest missingness among new cases.

Variable—Denominator	Bera & Sarkar [5]	New Cases	Combined
Auditory hallucinations—total-sample % (*n*)	8.3% (7/84)	12.5% (4/32)	9.5% (11/116)
Auditory hallucinations—available-case % (*n*)	9.0% (7/78)	23.5% (4/17)	11.6% (11/95)
Perceived foetal movement—total-sample % (*n*)	45.2% (38/84)	46.9% (15/32)	45.7% (53/116)
Perceived foetal movement—available-case % (*n*)	52.8% (38/72)	93.8% (15/16)	60.2% (53/88)
Drug-naive status—total-sample % (*n*)	35.7% (30/84)	25.0% (8/32)	32.8% (38/116)
Drug-naive status—available-case % (*n*)	38.5% (30/78)	50.0% (8/16)	40.4% (38/94)

*Note*. Total-sample % uses the full subsample (*n* = 84, 32, or 116) as denominator; available-case % excludes cases coded “not specified” for that variable. *n* in parentheses gives the numerator/denominator used for each percentage.

## Data Availability

The new data generated for this update—the complete case-level extraction dataset for the 32 newly identified cases, coded across all variables listed in Section 2.4 and pooled with the aggregate historical data published by Bera and Sarkar [5]—are provided in full in Table S1a,b (Supplementary Materials). No additional raw data beyond what is reported in the manuscript and the Supplementary Materials were generated or are available.

## Supplementary Materials

The following supporting information can be downloaded at https://www.mdpi.com/article/10.3390/jcm15197403/s1, Table S1a: Complete demographic and clinical/treatment data for the 32 new cases; Table S1b: Complete aetiological factor data for the 32 new cases; Table S2: Operational coding definitions for interpretive etiological and clinical categories; Table S3: PRISMA 2020 Checklist: completed reporting checklist for this review.
