# Supplementary material for: Delusion of Pregnancy: An Updated Systematic Review
Source: J Clin Med. 2026 Sep 23;15(19):7403. doi: 10.3390/jcm15197403 (PMC13665419; doi:10.3390/jcm15197403)
Supplement: Supplementary file 1 [file jcm-15-07403-s001.zip › jcm-4522256-supplementary.pdf]

Supplementary Materials

Delusion of Pregnancy: An Updated Systematic Review

Complete case-level data-extraction matrix for the 32 newly identified cases (September 2014 – August 2026), integrated with Bera and Sarkar's (2015) case series to form the combined sample (n = 116) reported in Tables 2–5 of the main text. All variables listed in §2.4 (Data Extraction) are reported here at the individual-case level, using the same categories applied in the aggregated analysis, so that the classification underlying every summary statistic in the manuscript can be independently audited. N/S = not specified in the source publication.

Table S1a

Demographic and clinical/treatment characteristics of the 32 new cases

ID	Author(s), Year	Country	Age	Sex	Marital status	Primary diagnosis	Duration	Conviction	N. foetuses	Auditory hall.	Foetal move.	Treatment
1	AlZamil, Alkhalifa & Azzoni, 2018	Saudi Arabia	39	M	Married	Comorbid psych.-medical	<3 mo	Delusional	Twin	Yes	N/S	Full
2	Arora et al., 2025 – Case 1	India	43	F	Married	Depressive	<3 mo	Delusional	Single	N/S	Yes	Partial
3	Arora et al., 2025 – Case 2	India	60	F	Married	Organic	<3 mo	Delusional	Single	N/S	Yes	Partial
4	Biswas & Saha, 2025	India	50	F	Married	Schizophrenia	3mo–1y	Delusional	Single	N/S	Yes	Full
5	Chag, Jadav, Chaudhury & Saldanha, 2023	India	57	F	Married	Depressive	1–2y	Delusional	Single	N/S	Yes	Full
6	Mehan, Wehner, Oberdorfer & Khalid, 2025	USA	64	M	Married	Schizophrenia	<3 mo	Delusional	Single	No	Yes	Full
7	Sethi & Vankar, 2021	India	43	F	Unmarried	Schizophrenia	1–2y	Delusional	Triplet	No	Yes	Partial
8	Sharma, Singh, Sahu & Pruthi, 2024 – Case 1	India	48	F	Married	Schizophrenia	3mo–1y	Delusional	Quadruplet+	No	N/S	Full
9	Sharma, Singh, Sahu & Pruthi, 2024 – Case 2	India	24	F	Married	Other psychotic	<3 mo	Delusional	Single	Yes	N/S	Partial
10	Sharma, Singh, Sahu & Pruthi, 2024 – Case 3	India	72	F	Separated/divorced/widowed	Organic	<3 mo	Delusional	Single	N/S	N/S	Partial
11	Sisek-Šprem et al., 2015 – Case A	Croatia	49	F	Separated/divorced/widowed	Comorbid psych.-medical	<3 mo	Delusional	Single	Yes	Yes	N/S
12	Sisek-Šprem et al., 2015 – Case B	Croatia	51	F	Unmarried	Organic	Not specified	Delusional	Quadruplet+	N/S	N/S	N/S
13	Sisek-Šprem et al., 2015 – Case C	Croatia	40	F	Married	Schizophrenia	Not specified	Delusional	Single	N/S	Yes	N/S
14	Sisek-Šprem et al., 2015 – Case D	Croatia	26	F	Unmarried	Schizophrenia	<3 mo	Delusional	Single	N/S	N/S	N/S
15	Sisek-Šprem et al., 2015 – Case E	Croatia	50	F	Separated/divorced/widowed	Schizophrenia	5–10y	Delusional	Quadruplet+	N/S	N/S	N/S
16	Sisek-Šprem et al., 2015 – Case F	Croatia	43	F	Separated/divorced/widowed	Comorbid psych.-medical	>10y	Delusional	Twin	N/S	N/S	N/S
17	Sisek-Šprem et al., 2015 – Case G	Croatia	48	F	Unmarried	Schizophrenia	Not specified	Delusional	Single	N/S	N/S	N/S
18	Tezcan & Turan, 2026	Turkey	39	F	Separated/divorced/widowed	Schizophrenia	3mo–1y	Delusional	Quadruplet+	N/S	N/S	Partial
19	Ursumando et al., 2022 – Case 1	Italy	29	F	Not specified	Other psychotic	3mo–1y	Delusional	Single	N/S	Yes	Full

ID	Author(s), Year	Country	Age	Sex	Marital status	Primary diagnosis	Duration	Conviction	N. foetuses	Auditory hall.	Foetal move.	Treatment
20	Ursumando et al., 2022 – Case 2	Italy	17	F	Not specified	Other psychotic	3mo–1y	Delusional	Single	N/S	Yes	N/S
21	Uvais, 2020	India	76	F	Separated/divorced/widowed	Organic	3mo–1y	Delusional	Single	N/S	N/S	N/S
22	Gaikwad, Dharmadhikari, Subramanyam, Kedare & Kamath, 2017	India	70	M	Married	Other	3mo–1y	Delusional	Single	No	N/S	Full
23	Goyal, Mahapatra, Goyal & Khandelwal, 2017	India	49	F	Married	Schizophrenia	1–2y	Delusional	Triplet	Yes	N/S	Full
24	Mascarenhas & Crasta, 2019	India	31	F	Married	Schizophrenia	3mo–1y	Delusional	Single	No	Yes	Partial
25	Tascon Guerra, Díaz Marqués & Manrique Ovejero, 2021	Spain	49	F	Unmarried	Other	3mo–1y	Delusional	Single	No	Yes	Full
26	Guilfoyle, O'Brien & O'Keeffe, 2015 – Case 1	Ireland	89	F	Not specified	Organic	<3 mo	Delusional	Single	No	No	Full
27	Guilfoyle, O'Brien & O'Keeffe, 2015 – Case 2	Ireland	74	F	Not specified	Organic	<3 mo	Delusional	Single	No	Yes	Full
28	Guilfoyle, O'Brien & O'Keeffe, 2015 – Case 3	Ireland	77	F	Not specified	Organic	<3 mo	Delusional	Single	No	N/S	Full
29	Guilfoyle, O'Brien & O'Keeffe, 2015 – Case 4	Ireland	86	F	Not specified	Organic	<3 mo	Delusional	Single	No	N/S	Full
30	Guilfoyle, O'Brien & O'Keeffe, 2015 – Case 5	Ireland	80	F	Not specified	Organic	<3 mo	Delusional	Single	No	N/S	Full
31	Suresh Kumar, Gopalakrishnan & Muhammed Farooque, 2016	India	52	M	Married	Other	3mo–1y	Delusional	Single	No	Yes	Full
32	Kumar, Zeeshan, Agrawal & Chaudhury, 2026	India	44	F	Married	Other	3mo–1y	Delusional	Single	No	Yes	Full

Table S1b

Aetiological factors of the 32 new cases (same case order as Table S1a)

ID	Author(s), Year	Psychosocial factors	Coenaesthopathic	Socio-cultural factors	Drug-naive	Hyperprolactin.
1	AlZamil, Alkhalifa & Azzoni, 2018	Wish-fulfilment	Yes	Not implicated	No	No
2	Arora et al., 2025 – Case 1	Wish-fulfilment	No	Not implicated	N/S	No
3	Arora et al., 2025 – Case 2	Wish-fulfilment	No	Not implicated	N/S	No
4	Biswas & Saha, 2025	Other	Yes	Not implicated	No	No
5	Chag, Jadav, Chaudhury & Saldanha, 2023	Loss of loved object	No	Societal pressure	N/S	No
6	Mehan, Wehner, Oberdorfer & Khalid, 2025	Other	No	Not implicated	No	No
7	Sethi & Vankar, 2021	Wish-fulfilment	No	Not implicated	N/S	No
8	Sharma, Singh, Sahu & Pruthi, 2024 – Case 1	Other	No	Other	No	No
9	Sharma, Singh, Sahu & Pruthi, 2024 – Case 2	Other	No	Other	Yes	Yes
10	Sharma, Singh, Sahu & Pruthi, 2024 – Case 3	Other	No	Not implicated	N/S	No
11	Sisek-Šprem et al., 2015 – Case A	Loss of loved object	Yes	Not implicated	No	Yes
12	Sisek-Šprem et al., 2015 – Case B	Loss of loved object	No	Not implicated	N/S	Yes
13	Sisek-Šprem et al., 2015 – Case C	Loss of loved object	No	Other	N/S	No
14	Sisek-Šprem et al., 2015 – Case D	Disturbed family dynamics	No	Not implicated	N/S	Yes
15	Sisek-Šprem et al., 2015 – Case E	Loss of loved object	No	Not implicated	N/S	Yes
16	Sisek-Šprem et al., 2015 – Case F	Loss of loved object	No	Not implicated	N/S	Yes
17	Sisek-Šprem et al., 2015 – Case G	Wish-fulfilment	No	Not implicated	N/S	No
18	Tezcan & Turan, 2026	Loss of loved object	No	Not implicated	No	Yes
19	Ursumando et al., 2022 – Case 1	Not implicated	Yes	Not implicated	Yes	Yes
20	Ursumando et al., 2022 – Case 2	Strong identification	Yes	Not implicated	Yes	No
21	Uvais, 2020	Wish-fulfilment	No	Not implicated	Yes	No
22	Gaikwad, Dharmadhikari, Subramanyam, Kedare & Kamath, 2017	Other	No	Not implicated	Yes	No
23	Goyal, Mahapatra, Goyal & Khandelwal, 2017	Wish-fulfilment	Yes	Other	No	Yes
24	Mascarenhas & Crasta, 2019	Other	Yes	Societal pressure	No	Yes
25	Tascon Guerra, Díaz Marqués & Manrique Ovejero, 2021	Other	No	Not implicated	Yes	No
26	Guilfoyle, O'Brien & O'Keeffe, 2015 – Case 1	Other	Yes	Not implicated	N/S	No
27	Guilfoyle, O'Brien & O'Keeffe, 2015 – Case 2	Not implicated	Yes	Not implicated	N/S	No
28	Guilfoyle, O'Brien & O'Keeffe, 2015 – Case 3	Not implicated	Yes	Not implicated	N/S	No

ID	Author(s), Year	Psychosocial factors	Coenaesthopathic	Socio-cultural factors	Drug-naive	Hyperprolactin.
29	Guilfoyle, O'Brien & O'Keeffe, 2015 – Case 4	Not implicated	Yes	Not implicated	N/S	No
30	Guilfoyle, O'Brien & O'Keeffe, 2015 – Case 5	Not implicated	Yes	Not implicated	N/S	No
31	Suresh Kumar, Gopalakrishnan & Muhammed Farooque, 2016	Sexual identity/abuse	Yes	Not implicated	Yes	No
32	Kumar, Zeeshan, Agrawal & Chaudhury, 2026	Not implicated	Yes	Not implicated	Yes	No

Table S2

Operational coding definitions for interpretive etiological and clinical categories

The categorical structure below follows Bera and Sarkar (2015), replicated for direct comparability with the original review. For each interpretive category, coding required either an explicit equivalent label in the source report or a clinical narrative judged clearly consistent with the definition given, in the absence of an equally or more consistent alternative category. Categories not listed here (e.g., sex, age, duration, treatment response) were coded directly from explicitly reported information and are not considered interpretive in the same sense; the operational rule for treatment-response coding specifically is given in §2.4 of the main text.

Category	Operational definition
Psychosocial factors (mutually exclusive; coded as the single best-fitting category)	
Wish-fulfilment	An explicitly stated or clearly inferable unfulfilled desire for a child or pregnancy (e.g., infertility, prior pregnancy loss, desire for a son/daughter) preceding onset of the delusion.
Loss of a loved object/relationship	Onset of the delusion is temporally and narratively associated with a significant loss (bereavement, relationship breakdown, separation, divorce).
Rebirth fantasies	Delusional content explicitly linked, by the patient or the original authors, to a wish to "restore" or symbolically replace a deceased or lost person through pregnancy.
Disturbed family dynamics	Family conflict, dysfunction, or estrangement is explicitly described as contextually related to symptom onset, without a clearer fit to another category.
Sexual abuse/sexual identity confusion	A history of sexual trauma, or documented conflict regarding sexual identity/orientation, is described as related to the delusional content.
Over-identification/rivalry/projection	The delusion is explicitly linked to identification with, or rivalry toward, a specific pregnant person in the patient's life (e.g., a sister, friend, or relative).
Strong identification with pregnant women	A general identification with pregnancy or motherhood as a valued social role is described, without an explicit rivalry or projection component.
Parental deprivation	Early loss of, or separation from, a parent is described as contextually relevant to symptom onset.
Intense ambivalence toward pregnancy	Explicit approach–avoidance conflict regarding the idea of pregnancy is described, without a clear unfulfilled-desire narrative.
Other	A psychosocial factor is clearly identified in the source report but does not fit any category above.
Not implicated	No psychosocial factor is identified or described in the source report (absence of textual evidence; see §4.4 for the interpretive caveat this implies).
Coenaesthopathic processes	
Yes	The source report describes the patient attributing delusional significance to a real, physiologically based bodily sensation (e.g., abdominal distension, constipation, altered menstrual bleeding, perceived movement) as evidence of pregnancy, consistent with the classical psychopathological construct of coenaesthopathy.

Category	Operational definition
No	No such sensation-based narrative is described, or the delusion is reported as arising without a stated bodily trigger.
Socio-cultural factors (mutually exclusive; coded as the single best-fitting category)	
Excessive societal pressure to procreate	Explicit cultural, familial, or social pressure to bear children is described as contextually relevant.
Chronic social deprivation	Prolonged social isolation or deprivation is described as contextually relevant (distinct from an acute, time-limited precipitating event, which is instead discussed narratively in §4.3).
Supernatural beliefs/evil eye	Delusional content explicitly incorporates supernatural, spiritual, or culturally specific causal beliefs (e.g., possession, the evil eye).
Other	A socio-cultural factor is clearly identified but does not fit any category above.
Not implicated	No socio-cultural factor is identified or described in the source report.
Drug-naive status at onset	
Yes	The source report explicitly states the patient had received no antipsychotic or other relevant psychotropic medication prior to symptom onset.
No	Prior psychotropic medication use is documented in the source report.
Not specified	Medication history prior to onset is not reported.
Hyperprolactinaemia	
Yes	An elevated prolactin level is explicitly reported (laboratory-confirmed) in the source report.
No	Prolactin is either reported as within normal limits or not mentioned in the source report (see §4.4 for the interpretive caveat this binary coding implies).
Primary diagnosis — selected categories requiring clarification	
Organic	The source report attributes the delusion to a medical/neurocognitive condition (e.g., dementia, delirium, metabolic disturbance) in the absence of a separate, formally diagnosed psychiatric disorder.
Comorbid psychiatric and medical	The source report documents both a formal psychiatric diagnosis (e.g., schizophrenia, bipolar disorder) and a concurrent organic/medical condition explicitly described by the original authors as contributing to, or co-occurring with, the delusional presentation (e.g., epilepsy, tuberous sclerosis).

PRISMA 2020 Checklist

Completed following Page MJ, McKenzie JE, Bossuyt PM, Boutron I, Hoffmann TC, Mulrow CD, et al. The PRISMA 2020 statement: an updated guideline for reporting systematic reviews. BMJ 2021;372:n71.

Section	Item #	Checklist item	Location / reporting in this manuscript
**TITLE**			
Title	1	Identify the report as a systematic review.	Title page — "Delusion of Pregnancy: An Updated Systematic Review"
**ABSTRACT**			
Abstract	2	See the PRISMA 2020 for Abstracts checklist.	Abstract, p. 1
**INTRODUCTION**			
Introduction	3	Describe the rationale for the review in the context of existing knowledge.	§1 Introduction, particularly the paragraphs summarising the state of the art and the rationale for the update
Introduction	4	Provide an explicit statement of the objective(s) or question(s) the review addresses.	§1, "The Aim of the Study" paragraph (end of Introduction)
**METHODS**			
Methods	5	Specify the inclusion and exclusion criteria for the review and how studies were grouped for the syntheses.	§2.3 Inclusion and Exclusion Criteria
Methods	6	Specify all databases, registers, websites, organisations, reference lists and other sources searched, and the date of the last search for each source.	§2.2 Search Strategy; Table 1 (search date per database)
Methods	7	Present the full search strategies for all databases, registers and websites, including filters and limits used.	Table 1 (verbatim search strings, filters, dates)
Methods	8	Specify the methods used to decide whether a study met the inclusion criteria, including how many reviewers screened each record/report, whether screening was independent, and if applicable, details of automation tools.	§2.4 Study Selection and Data Extraction (two authors, independent, in duplicate; disagreements resolved by discussion; third reviewer available but not required)
Methods	9	Specify the methods used to collect data from reports, including how many reviewers collected data, whether collection was independent, any processes for obtaining/confirming data from investigators, and if applicable, details of automation tools.	§2.4 (data extraction independent and in duplicate by both authors)
Methods	10a	List and define all outcomes for which data were sought.	§2.4, items (b) clinical/treatment characteristics
Methods	10b	List and define all other variables for which data were sought.	§2.4, items (a) demographic and (c) aetiological characteristics
Methods	11	Specify the methods used to assess risk of bias in the included studies.	Not performed. No formal risk-of-bias or critical-appraisal tool (e.g., JBI checklists) was applied to individual case reports/series. This choice is explicitly declared in §2.4 and justified as a limitation in §4.4.

Section	Item #	Checklist item	Location / reporting in this manuscript
Methods	12	Specify the effect measure(s) used in presenting results.	Not applicable. No pooled effect measures were calculated; this update reports descriptive frequencies and percentages only (§2.5).
Methods	13a	Describe the processes used to decide which studies were eligible for each synthesis.	§2.5 Data Synthesis (all included cases entered the single descriptive synthesis)
Methods	13b	Describe any methods required to prepare the data for presentation or synthesis.	§2.4–2.5 (categorical extraction grid; summation of new counts with published 2015 counts)
Methods	13c	Describe any methods used to tabulate or visually display results of individual studies and syntheses.	Tables 2–5 (Results); Table S1 (Supplementary Materials)
Methods	13d	Describe any methods used to synthesize results.	§2.5 (descriptive summation and recalculation of percentages on the combined denominator, n = 116)
Methods	13e	Describe any methods used to explore possible causes of heterogeneity among study results.	Not applicable — no quantitative/meta-analytic synthesis was performed; qualitative discussion of between-subsample differences is provided in §3.2 and §4.3
Methods	13f	Describe any sensitivity analyses conducted to assess robustness of the synthesized results.	A sensitivity analysis was conducted excluding three borderline cases (Sisek-Šprem et al., 2015) from the new-case sample and recalculating all combined-sample statistics; see §2.5 and §3.2 (Table 6).
Methods	14	Describe any methods used to assess risk of bias due to missing results in a synthesis.	Not formally assessed. Publication bias favouring atypical presentations is discussed narratively as a limitation (§4.4).
Methods	15	Describe any methods used to assess certainty (or confidence) in the body of evidence for an outcome.	Not performed (e.g., no GRADE assessment); not considered applicable to a descriptive case-report synthesis.
**RESULTS**			
Results	16a	Describe the results of the search and selection process, from the number of records identified to the number of studies included, ideally using a flow diagram.	Figure 1 (PRISMA 2020 flow diagram); §2.2 narrative summary
Results	16b	Cite studies that might appear to meet the inclusion criteria, but which were excluded, and explain why they were excluded.	§2.3 (three full-text exclusions with individual reasons); Figure 1
Results	17	Cite each included study and present its characteristics.	Table S1 / Appendix A (Supplementary Materials): all 32 new cases individually cited with characteristics
Results	18	Present assessments of risk of bias for each included study.	Not applicable (no risk-of-bias assessment was performed; see Methods item 11 and §4.4 Limitations).
Results	19	For all outcomes, present, for each study: (a) summary statistics and (b) an effect estimate and its precision.	Not applicable in the meta-analytic sense; per-case data are reported in Table S1, and aggregated frequencies in Tables 2–5
Results	20a	Briefly summarise the characteristics and risk of bias among studies contributing to the synthesis.	§3.1–3.2; risk-of-bias summary not applicable (see item 11)
Results	20b	Present results of all statistical syntheses conducted.	Tables 2–5 (descriptive frequencies for the combined sample and old/new subsample comparison)

Section	Item #	Checklist item	Location / reporting in this manuscript
Results	20c	Present results of all investigations of possible causes of heterogeneity among study results.	Not applicable — addressed narratively in §4.3 (Discussion) rather than through formal heterogeneity statistics
Results	20d	Present results of all sensitivity analyses conducted to assess the robustness of the synthesized results.	Table 6 (§3.2): all five headline percentages examined changed by ≤2.1 percentage points when three borderline cases were excluded, indicating the combined-sample findings are not driven by these cases.
Results	21	Present assessments of risk of bias due to missing results for each synthesis assessed.	Not formally assessed; discussed narratively (§4.4)
Results	22	Present assessments of certainty in the body of evidence for each outcome assessed.	Not performed (see Methods item 15)
**DISCUSSION**			
Discussion	23a	Provide a general interpretation of the results in the context of other evidence.	§4 Discussion (opening paragraphs and §4.1–74.2)
Discussion	23b	Discuss any limitations of the evidence included in the review.	§4.4 Limitations
Discussion	23c	Discuss any limitations of the review processes used.	§4.4 Limitations (reporting heterogeneity, publication bias, lack of prospective registration)
Discussion	23d	Discuss implications of the results for practice, policy, and future research.	§4.2 Practical Implications; §4.3 Future Directions
**OTHER INFORMATION**			
Other	24a	Provide registration information for the review, including the register name and registration number, or state that the review was not registered.	Not registered. See §2.4 and §4.4: the review followed a predefined internal protocol that was not prospectively registered in a public registry such as PROSPERO.
Other	24b	Indicate where the review protocol can be accessed, or state that a protocol was not prepared.	Internal protocol; not deposited in a public repository. [Authors to confirm availability on request, if applicable.]
Other	24c	Describe and explain any amendments to information provided at registration or in the protocol.	Not applicable (no public protocol registration).
Other	25	Describe sources of financial or non-financial support for the review, and the role of the funders/sponsors in the review.	[Not currently stated in the manuscript — authors should confirm/insert a Funding statement, e.g., "This research received no external funding."]
Other	26	Declare any competing interests of review authors.	Conflicts of Interest statement (Title page/Declarations): authors declare no conflict of interest
Other	27	Report which of the following are publicly available: template data collection forms; data extracted from included studies; data used for all analyses; analytic code; any other materials used in the review.	Data Availability Statement (Declarations); full case-level extraction data provided in Table S1 (Supplementary Materials)
